# Re-Expression of IGF-II Is Important for Beta Cell Regeneration in Adult Mice

**DOI:** 10.1371/journal.pone.0043623

**Published:** 2012-09-07

**Authors:** Luxian Zhou, Stella Pelengaris, Sylvie Abouna, James Young, David Epstein, Julia Herold, Tim Wilhelm Nattkemper, Hassan Nakhai, Michael Khan

**Affiliations:** 1 School of Life Sciences, University of Warwick, Coventry, United Kingdom; 2 Warwick Medical School, University of Warwick, Coventry, United Kingdom; 3 Department of Mathematics, University of Warwick, Coventry, United Kingdom; 4 Biodata Mining Group, Bielefeld University, Bielefeld, Germany; University of Texas Health Science Center at San Antonio, United States of America

## Abstract

**Background:**

The key factors which support re-expansion of beta cell numbers after injury are largely unknown. Insulin-like growth factor II (IGF-II) plays a critical role in supporting cell division and differentiation during ontogeny but its role in the adult is not known. In this study we investigated the effect of IGF-II on beta cell regeneration.

**Methodology/Principal Findings:**

We employed an *in vivo* model of ‘switchable’ c-Myc-induced beta cell ablation, pIns-c-MycER^TAM^, in which 90% of beta cells are lost following 11 days of c-Myc (Myc) activation *in vivo*. Importantly, such ablation is normally followed by beta cell regeneration once Myc is deactivated, enabling functional studies of beta cell regeneration *in vivo*. IGF-II was shown to be re-expressed in the adult pancreas of pIns-c-MycER^TAM^/IGF-II^+/+^ (MIG) mice, following beta cell injury. As expected in the presence of IGF-II beta cell mass and numbers recover rapidly after ablation. In contrast, in pIns-c-MycER^TAM^/IGF-II^+/−^ (MIGKO) mice, which express no IGF-II, recovery of beta cell mass and numbers were delayed and impaired. Despite failure of beta cell number increase, MIGKO mice recovered from hyperglycaemia, although this was delayed.

**Conclusions/Significance:**

Our results demonstrate that beta cell regeneration in adult mice depends on re-expression of IGF-II, and supports the utility of using such ablation-recovery models for identifying other potential factors critical for underpinning successful beta cell regeneration *in vivo*. The potential therapeutic benefits of manipulating the IGF-II signaling systems merit further exploration.

## Introduction

Inadequate numbers of beta cells characterize essentially all major types of diabetes. Therefore, identifying means by which beta cells could be replaced or expanded in number is a major goal in diabetes research and would form an integral part of attempts to develop new, more effective treatments and ultimately to cure the disease. The most successful approach to date for increasing beta cell numbers is through transplantation, but limited supplies of donor islets will restrict this approach to a handful of individuals. Some encouraging therapeutic results have been obtained by administering thiazoledenediones or GLP-1 analogues to various diabetes models [1], [2] and also in man [3], [4]. However, those factors actually supporting beta cell regeneration in animal models *in vivo* have not been functionally analyzed to date. A new source of beta cells or a means of encouraging beta cell renewal endogenously would be of major importance, but will first require a significant increase in our basic understanding about how beta cell renewal is regulated in normal physiology and in disease.

Beta cell renewal may take place by differentiation of stem cells [5], [6] or by replication of existing differentiated cells, of the same [7] or of a different lineage [8]. The favored mechanism/s may be determined by the nature of the demand or initial injury [9]. Beta cell regeneration might require combinations of growth factors and metabolic effects as well as external stimuli [10]. Despite much progress, the key signals triggering beta cell regeneration *in vivo* have not been examined functionally and are still not fully understood. The insulin-like growth factor II gene (*igf2*) is imprinted with silencing of the maternal allele and found to be widely expressed during murine embryonic development and particularly important in placental growth [11], [12]. Concentrations of IGF-II in fetal plasma and tissues are high, but drop dramatically postnatally [11], [13]–[15]. In IGF-II KO mice, intra-uterine growth is retarded [16]. The normal increase in beta cell apoptosis in neonatal rats is associated with reduced IGF-II expression [17], [18] and in mice such apoptosis can be suppressed by increasing IGF-II. In the GK (Goto–Kakizaki) rat diabetes model, defective IGF-II and IGF-IR expression precede a decrease in beta cell mass, whereas IGF-II supplementation expands the pool of beta cells [19]. In the adult, *igf2* loss of imprinting (LOI) and re-expression of IGF-II have been shown in cancer [20], [21]. In an animal model, disruption of both IGF-II alleles reduced neoplastic growth of beta cells [22], [23]; this suggests that, at least under these conditions, the prevention of re-expression of IGF-II in the adult can restrict growth of neoplastic beta cells. Beta cell regeneration after injury has not been examined in such mice. Given the complex phenotypes observed in many of these earlier studies, it becomes particularly important to ascertain which factors might be critical in supporting beta cell regeneration *in vivo*. One of the only such studies to date has examined recovery of beta cell mass after partial pancreatectomy and has found this to be impeded by deletion of GLP-1R [24], [25].

To summarize, there is little doubt that various growth factors may be employed to enhance beta cell numbers or even to mitigate against the consequences of beta cell injury. However, the actual functional role played by endogenous growth factors during beta cell regeneration *in vivo* has received limited attention to date. This may at least in part be due to the absence until relatively recently of transgenic mice models in which ablation can be easily and reproducibly induced and from which beta cell numbers can recover. Such models are now available of which the pIns-c-MycER^TAM^ was the first [26], [27]. Research to date strongly indicates that IGF-II is important in regulating pancreatic beta cell mass during ontogeny, might be usefully deployed therapeutically in the adult, and is functionally important in beta cell neoplasia. However, whether IGF-II plays any role in regeneration of normal beta cells in the adult is not known and this question forms the basis of this study. Here for the first time we exploit one of the new conditional beta cell ablation models to study factors important for effective beta cell regeneration *in vivo*.

## Results

### IGF-II is re-expressed after brief activation of Myc in pIns-c-MycER^TAM^/IGF-II^+/+^ mice

**Figure 1:**
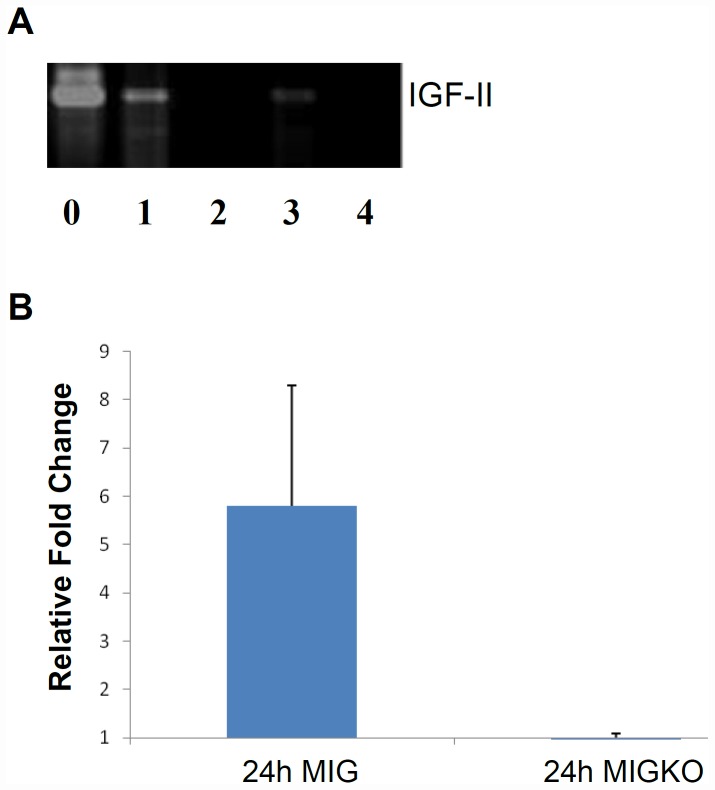
IGF-II re-expression in MIG mice after brief Myc activation. Total pancreas RNA was extracted from pIns-c-MycER^TAM^/IGF-II^+/+^ (MIG) and pIns-c-MycER^TAM^/IGF-II^+/−^ (MIGKO) mice at 24 hr and 48 hr following Myc activation to induce beta cell ablation (n = 3). RT-PCR data (A) shows the amplified products in MIG mice after 24 hr (1) and 48 hr (3) of Myc activation in the target size of 357 bp. No amplified product was found in MIGKO mice at 24 hr (2) and 48 hr (4) of Myc activation by comparing to the positive control (WT mouse E17.5 placenta RNA) (0). Quantitative RT-PCR was performed and validated the RT-PCR results. IGF-II mRNA was expressed 5-fold higher in MIG mice after brief Myc activation but not in MIGKO mice nor in negative controls (B).

Beta cell injury was induced as previously described [27], [28]. Briefly, beta cell apoptosis was induced in pIns-c-MycER^TAM^/IGF-II^+/+^ (MIG) and pIns-c-MycER^TAM^/IGF-II^+/−^ (MIGKO) mice by activating c-Myc (Myc) for up to 48 hours through daily intraperitoneal (IP) administration of 4-OHT (0.5 mg). 48 hours was chosen as beta cell injury is induced but overall islet mass has not yet decreased significantly, thus allowing maximum chance of detecting any islet re-expression of IGF-II. Total pancreas RNA was extracted and reverse transcribed to cDNA. By amplifying the target product we show that IGF-II is re-expressed after beta cell injury *in vivo* (Fig. 1A). As expected IGF-II mRNA was undetectable in MIGKO mice even after beta cell injury, whereas MIG mice expressed IGF-II mRNA, as we found by sequencing the product and comparing with the relevant database (data not shown). Also qRT-PCR was performed to measure the IGF-II mRNA expression level in these two strains. Our results confirmed re-expression of IGF-II mRNA in MIG mice after brief Myc activation (Fig. 1B). And again, as expected, we found no detectable IGF-II in MIGKO mice after Myc activation.

### Loss of IGF-II retards recovery of hyperglycemia following beta cell ablation

**Figure 2:**
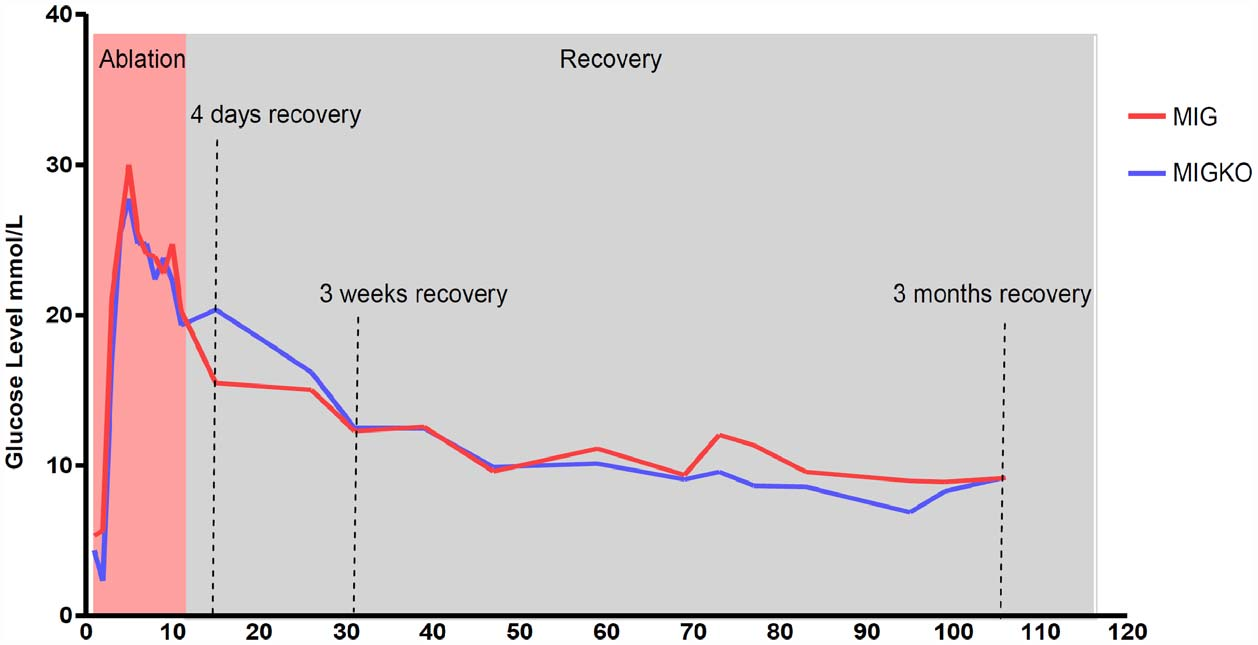
Blood glucose homeostasis. Myc activation was induced for 11 days in mice following which Myc was deactivated for up to 3 months. Blood glucose levels (Mean ± SEM) were measured in MIG mice n = 3 and MIGKO mice n = 3 during this period (A). Both strains developed hyperglyceamia after Myc activation and MIGKO mice showed a delay of recovery from hyperglycemia at day 4 following Myc deactivation. After 3 months both strains were able to return to normal blood glucose level.

**Figure 3:**
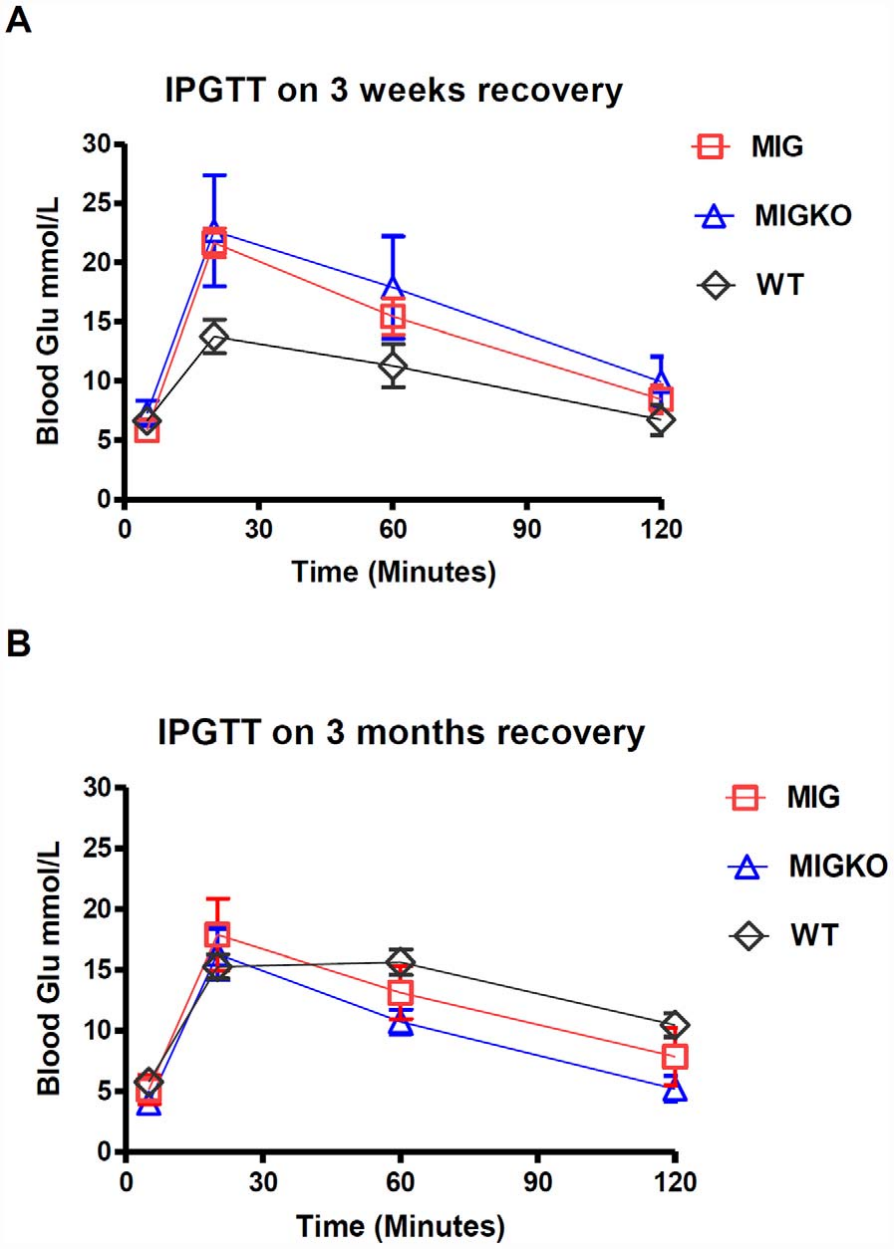
Intraperitoneal glucose tolerance test. Islet functional study was performed by taking IP glucose tolerance test (IPGTT). Mice were starved overnight before intraperitoneal injection of glucose at 1.5 mg/g of animal body weight. Results of IPGTT showed both stains did not recover 3 weeks after Myc deactivation, comparing to their wildtype litter mates (A). After 3 month recovery both strains were able to maintain normal blood glucose (B).

We have previously shown that activation of Myc in pancreatic beta cells of pIns-c-MycER^TAM^ mice, results in around 90% beta cell ablation and hyperglycemia [27]. In this study we show that, after 11 days of Myc activation in pIns-c-MycER^TAM^ mice, both wildtype for IGF-II (MIG) and IGF-II KO (MIGKO) mice developed hyperglycaemia. Similar blood glucose level was found for two strains before Myc activation: 5.3±0.6 mmol/L for MIG mice (n = 10) and 4.4±0.5 mmol/L for MIGKO mice (n = 10) (p = 0.1900). After activation of Myc both strains developed hyperglycaemia within 4 days which persisted throughout the treatment period (Fig. 2). The difference in peak glucose levels between MIG and MIGKO mice was not significant: (30.0±0.8 mmol/L vs. 27.6±1.3 mmol/L; p = 0.1255) (n = 9). After deactivating Myc (withdrawal of 4-OHT) for 4 days MIG mice started to recover from hyperglycaemia (15.5±4.8 mmol/L; n = 3), which was not however, observed in MIGKO mice (20.4±4.4 mmol/L; n = 3). However, over time both strains achieve normal blood glucose levels and there were no detectable differences after 3 months of recovery. An intraperitoneal glucose tolerance test (IPGTT) was performed at different recovery time points to explore glucose homeostasis in more detail. As expected and previously shown, control mice had normal glucose tolerance tests while neither MIG nor MIGKO mice were able to maintain normal blood glucose 3 weeks after Myc deactivation (Fig. 3A). Both strains had recovered after 3 months (Fig. 3B). However, recovery of a normal IPGTT at 3 months does not imply that beta cell mass has recovered, since as few as 10% to 20% of the normal number of functional beta cells could suffice for this [29].

### Loss of IGF-II retards recovery of beta cell mass following beta cell ablation

The beta cell mass of both MIG and MIGKO mice was examined prior to and after Myc activation. Pancreata were carefully sectioned at 100 µm intervals and sections randomly selected from at least 5 different levels through the length of the pancreas. Following 11 days of Myc activation beta cell mass in MIG and MIGKO mice lost around 90% of normal pre-ablation mass, with only 0.407±0.058 mg and 0.300±0.238 mg left, respectively (Table S1 for raw data). Within 4 days of deactivation of Myc (recovery), beta cell mass had already increased in MIG mice. In MIGKO mice there was no such increase, though there was some recovery of beta cell mass by 3 months after ablation.

**Figure 4:**
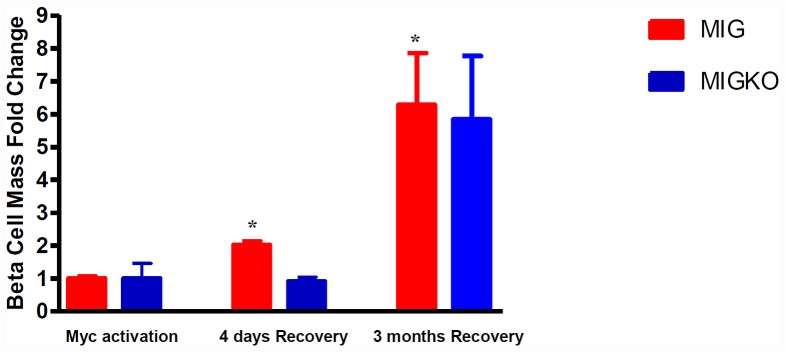
Loss of IGF-II impedes recovery of beta cell mass following beta cell ablation. For each individual, beta cell mass was calculated as cross sectional area of insulin immunoreactivity divided by total sectional area for at least 5 levels (n = 3). Results showed in MIG mice beta cell mass increased 2-fold at 4 days Myc deactivation (p = 0.0106), whereas in MIGKO mice the beta cell mass was largely unchanged. After 3 months recovery the beta cell mass increased 6-fold in MIG mice and in MIGKO mice the increase was 5-fold.

In order to assess and compare the ability to recover from beta cell ablation in the presence or absence of IGF-II, we have measured the increase in beta cell mass after ablation. At 4 days recovery, beta cell mass in MIG mice had already increased 2-fold from post-ablation levels (p = 0.0106). In MIGKO mice no increase in beta cell mass was noted (Fig. 4). After 3 months post-ablation beta cell mass in MIG mice had increased more than 6-fold compared to immediate post-ablation mass (p = 0.0339). In MIGKO mice there was also an apparent increase by 3 months, but this did not reach the statistical significance (p = 0.0600) (Fig. 4).

### Loss of IGF-II retards recovery of beta cell numbers following beta cell ablation

As cell mass and cell number might not necessarily correlate (changes in cell size could affect the former), we estimated numbers of insulin positive beta cells in five pancreas sections per mouse. The counting was performed manually and also by the application of new machine based learning software co-developed and validated with our collaborators [30]. The data was largely consistent with that obtained for beta cell cross sectional area and mass. Consistently with beta cell mass data, beta cell numbers dropped by 84.7% in MIG and 87.0% in MIGKO mice following ablation (Table S2 for raw data).

**Figure 5:**
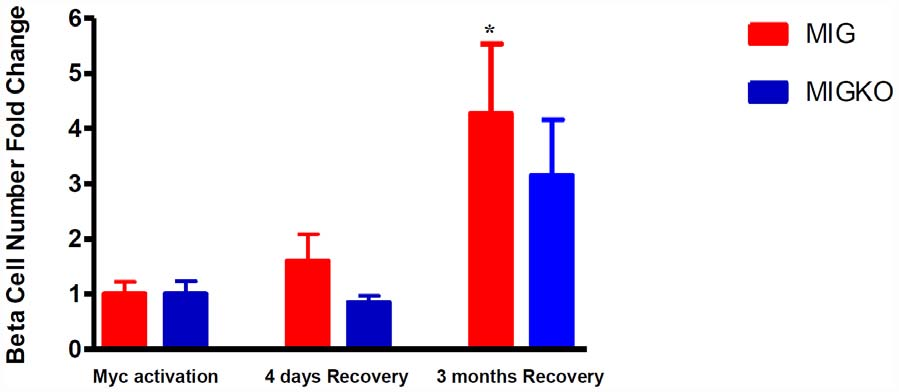
Loss of IGF-II affects recovery of beta cell numbers following beta cell ablation. Beta cell number was counted in up to 200 islets from five pancreas levels per mouse (n = 3) by software co-developed with our collaborators [30]. Results showed in MIG mice beta cell number increased by 60% at 4 days Myc deactivation, whereas in MIGKO mice the beta cell mass was barely changed. Consistently with our previous observation in beta cell mass after 3 months recovery, both MIG and MIGKO mice achieved 3- to 4-fold increase of beta cell numbers.

Recovery of beta cell numbers following ablation was assessed in the two strains to assess and compare the ability to recover from beta cell ablation in the presence or absence of IGF-II. At 4 days recovery, beta cell number in MIG mice had already increased by around 60% from post ablation levels, whereas, as noted for beta cell mass, there was no change in beta cell numbers in MIGKO mice (Fig. 5).

After 3 months, beta cell numbers in MIG mice had increased more than 4-fold from numbers post ablation (p = 0.0466). A trend towards recovery was noted in MIGKO nearly reaching statistical significance (p = 0.0557) (Fig. 5).

## Discussion

There are multiple mechanisms involved in beta cell renewal [5]–[8], [31] and much effort has been devoted to identifying those growths factors that can potentially promote and support beta cell regeneration. However, the key signals triggering beta cell replication and regeneration still remain to be discovered. Many growth factors have been shown to increase beta cell regeneration and suppress apoptosis [32], [33], but few have been shown to be essential for beta cell regeneration, suggesting considerable redundancy in signalling. The Insulin-IGF signaling pathway has received particular attention in recent decades. Expression of IGF-I in beta cells has been shown to protect transgenic mice from STZ-induced diabetes, partially by promoting beta cell replication and neogenesis [34]. Employing the double mutant mice lacking *Igf1r* specifically in pancreatic beta cells in an *Irs1*- or *Irs2*-null background, Dr. Accili's group recently demonstrated that beta cell proliferation is mainly regulated by *InsR* using *Irs2* as a downstream signaling effector [35]. The receptors for insulin/IGFs have also been examined functionally using beta cell-specific IR and IGF-IR knockout mice (βIRKO, βIGF-IRKO). Neither the βIRKO nor the βIGF-IRKO mice show any changes in islet mass at least in the first two months of life, after which the βIRKO mice show a decline in islet mass probably as a result of functional defects in the islet and the onset of diabetes [36], [37]. The insulin-like growth factor-II gene (*igf2*) is one member of this family and is widely expressed during murine embryonic development. It is particularly important in placental growth [11], [12]. IGF-II is expressed in the endocrine tissue of the pancreas during fetal and early neonatal stages in rats and humans. In the adult, IGF-I expression predominates and after day 7 in neonatal rats is expressed in the ductal and acinar tissue and its expression increases with age [11], [13]–[15]. A potential role for IGF-II function in the pancreas is supported by two bodies of work; those showing a key functional role for loss of imprinting and re-expression of IGF-II in supporting the expansion and progression of beta cell-derived cancers [20], [21] and those showing the activity of IGF-II as a survival factor, *in vitro* and *in vivo*, largely preventing the wave of developmental apoptosis [17], [18] and having an effect on beta cell mass regulation [19], [38]. However, as very few studies have examined the functional role of endogenous factors in beta cell regeneration, and in particular no such studies had examined the role of IGF-II in this context, we set out to employ a regulatable transgenic mouse model pIns-c-MycER^TAM^, which usually exhibits remarkable beta cell regeneration after ablation [27], to investigate the function of IGF-II in this process.

We demonstrate for the first time that IGF-II expression is reactivated after beta cell injury. We detected IGF-II mRNA at 24 hr and 48 hr after initiation of beta cell injury in MIG mice but as expected not in MIGKO mice, which do not express IGF-II. Critically, when the re-expression of IGF-II is prevented, by knock-out of the non-imprinted allele, then the recovery of beta cell mass and numbers are delayed and substantially reduced.

We find MIGKO mice start with fewer beta cells and lower beta cell mass compared to MIG mice before ablation but this is proportionate to their smaller size and body weight. As expected, Myc activation leads to extensive beta cell ablation in both MIG as well as in MIGKO mice, suggesting that the re-expression of IGF-II following Myc-induced beta cell injury does not prevent Myc induced apoptosis (this is not surprising as even administration of large doses of growth factors, such as insulin and GLP-1 analogues fails to slow this [28] (Young *et al.* unpublished observation). However, the re-expression of IGF-II appears to be very important for the early recovery (4 days) of beta cell mass, which was also indicated by retarded recovery from hyperglycaemia in MIGKO mice. Even though for experimental reasons we have not been able to confirm the continued long-term re-expression of IGF-II, our results support the contention that re-expression of IGF-II affects early beta cell regeneration. At the longer time points (3 months) IGF-II seems to be dispensable as both MIG and MIGKO mice displayed increase in beta cell mass/number and showed no statistical/biological differences. It is possible that other growth factors such as IGF-1 could play a critical role in this process.

**Figure 6:**
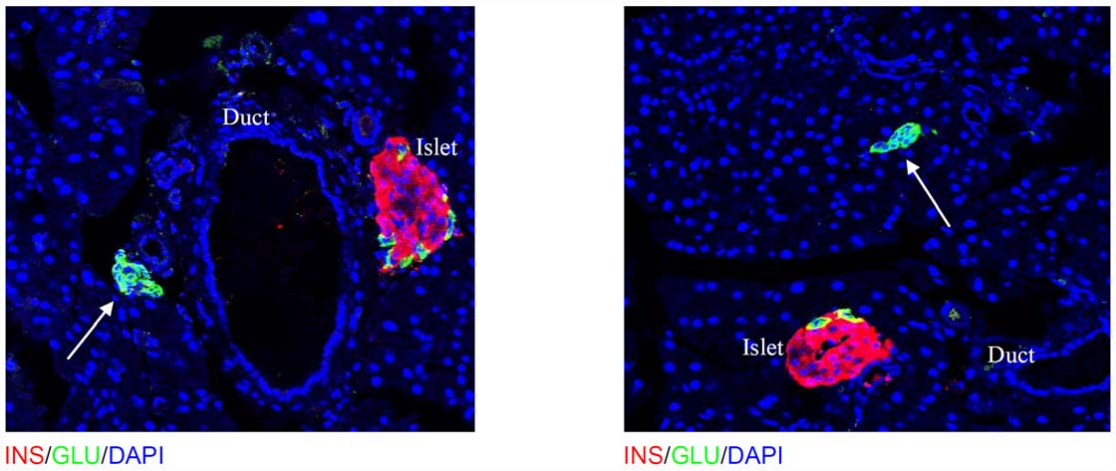
Glucagon positive cell cluster in MIG mice after 3 months Myc deactivation. In MIG mice after 3 months of recovery, clusters of glucagon positive cells separate from islets were observed. This might be new islets forming or possible clusters of alpha cells which might then further differentiate into beta cells. Such clusters were not seen in MIGKO mice or control mice pre-ablation.

In the adult, new beta cells may arise by a combination of differentiation from stem cell precursors [5], [6] or by replication of existing differentiated cells [7], [8]. A very recent study suggested that pancreatic-duct cells serve as a partial source of regeneration [39]. It is also possible that differences in beta cell mass could be the result of modulation of rates of apoptosis. An intriguing study showed that alpha cells can transdifferentiate to beta cells after extreme beta-cell loss [40]. Another study suggested a period of quiescence from beta cell replication may be controlled by glucose and aging [41]. Also recent work from our group has shown that a substantial proportion of new beta cells arise from a non-beta cell source, such as a stem cell progenitor, during pregnancy [42]. We have also therefore determined the rate of beta cell proliferation in MIG and MIGKO mice after 3 months recovery. However, no difference was detected between the two strains (data not shown). One explanation could be that much of the replication takes place earlier, but how likely this is given the fact that beta cell mass/numbers have still not fully recovered to pre-ablation levels is debatable. We did not observe any difference between MIG and MIGKO in beta cell apoptosis at 3 months recovery. However, in MIG mice after 3 months we observed some clusters of cells separate from islets in which only glucagon positive cells could be seen. Although, this might be the result of total beta cell ablation leaving only alpha cells behind the same phenomenon was not observed in MIGKO mice (Fig. 6). It will be interesting in future to determine whether these could be a source of new beta cells by trans-differentiation. More studies would be needed to address the source of regenerating beta cells between MIG and MIGKO mice. One possibility would be to further cross these mice into reporter strains for lineage tracking [7], [42].

To date we have not been able to determine the exact cellular source of IGF-II in the pancreas. The strong presence of mRNA for IGF-II supports the notion that this lies within the pancreas, but we have not been able to successfully or reliably localize peptide expression to any given cell type using immunohistochemistry. The difficulty is further compounded by the unavailability of an antibody that works in Western blots for IGF-II peptide, as previously noted by other groups. It is hoped that *in situ* hybridization may help resolve this issue and we are pursuing this matter further at the present time.

It is also important to note that these studies reveal the utility of conditional beta cell ablation models for undertaking functional studies of beta cell regeneration and it is anticipated that many more such will follow so that a more complete picture can be drawn of those factors essential for beta cell regeneration *in vivo*. Such factors, which now include IGF-II, are of obvious therapeutic interest as they may be exploitable as new therapeutic agents in the treatment or even prevention of diabetes.

## Materials and Methods

### Transgenic mice

*pIns-c-MycER^TAM^* was generated by cloning a full-length human c-*Myc* cDNA fused to the hormone-binding domain of a modified estrogen receptor (c-MycER^TAM^) downstream of the rat insulin promoter as previously described [27]. IGF-II KO mice were a generous gift from Professor Doug Hanahan, UCSF [22]. Since *igf2* is an imprinted gene with the maternal allele silenced [11], IGF-II KO mice are first identified by small size and the genotype confirmed as previously described [30]. IGF-KO strain is crossed with *pIns-c-MycER^TAM^* mice to generate pIns-c-MycER^TAM^/IGF-II^+/+^ (MIG) and pIns-c-MycER^TAM^/IGF-II^+/−^ (MIGKO) mice.

Transgenic mice were housed under barrier conditions with a 12 hour light/dark cycle and access to food and water.

### Experimental Procedure

This study was carried out in strict accordance with the recommendations in the Guide for the Care and Use of Laboratory Animals of the National Institutes of Health. The protocol was approved by the Ethics Committee of the University of Warwick and covered by a UK HO project licence.

Myc activation in groups of MIG and MIGKO mice aged around 3 months old was induced by daily administration of 4-hydroxytamoxifen (4-OHT) or tamoxifen (Sigma) as previously described [27], [43]. Inactivation of c-MycER^TAM^ protein was achieved following withdrawal of the drug [27]. Hence, to quantify beta cells, MIG (n = 3) and MIGKO (n = 3) mice were injected for 11 days and then groups of mice were either culled immediately or after 4 days or after 3 months following cessation of Myc activation. To detect IGF-II mRNA re-expression, 3 months old male mice from both MIG (n = 3) and MIGKO (n = 3) were injected with 0.5 mg 4-OHT up to 48 hr to induce beta cell injury. Non-fasted blood glucose concentrations were sampled regularly from tail bleeds using an Advantage glucose meter (Roche Diagnostics, Burgess Hill, UK).

A glucose tolerance test for the two strains was performed at 3 weeks recovery and at 3 months recovery. Mice were starved overnight and intraperitoneal injected 1.5 mg glucose per gram of mouse weight. Blood glucose was monitored prior to and at 20, 60 and 120 minutes after glucose administration using an Advantage glucose meter (Roche Diagnostics, Burgess Hill, UK).

### RNA extraction and Reverse Transcription

The pancreata were collected at the end of the experiment and immediately frozen in liquid nitrogen. Placenta from wildtype mouse was used to extract RNA as positive control. Then total RNA was isolated and stabilized by homogenizing the pancreas in RLT buffer (with beta-mercaptoethanol) for 1 min. Then the total RNA was extracted and purified by the RNA Extracting mini Kit (Qiagen) and DNase Kit (Qiagen). The purified RNA was tested by Nanodrop spectrophotometer to estimate the concentration. The RNA of each pancreas was reverse transcripted to get the cDNA using Superscrip-II kit (Invitrogen). IGF-II PCR was performed with the following primers: IGF2 F: 3′- GTG CGG AGG GGA GCT TGT TGA C-5′; IGF2 R: 5′-GTG GGC GTC TTT GGG TGG TA-3′ PCR product size: 357 bp.

The cDNA from the gel was collected, extracted and purified by the extracting kit from Qiagen. The IGF-II cDNA was sequenced by Molecular Biology Service, Biological Department, University of Warwick.

### Quantitative Real-time Reverse Transcription Polymerase Chain Reaction (qRT-PCR)

qRT-PCR was set up on a 96-well PCR plate. For each well, the PCR master mix consisted of 10 µl TaqMan 2× gene-expression Master Mix, 9 µl cDNA sample and 1 µl TaqMan qRT-PCR gene-expression assay or 18 s rRNA control. Individual gene-expression assays, 18 s rRNA endogenous positive control probe (Applied Biosystems, Foster City, CA) and water negative control were run in triplicate wells. qRT-PCR was performed using an ABI Prism 7000 scanner (Applied Biosystems, Foster City, CA), using the following program: 2 min at 50°C for activation of Uracil-DNA glycosylase, 10 minutes at 95°C for activation of AmpliTaq Gold enzyme and 40 cycles of 95°C for 15 seconds and 60°C for 1 minute.

### Histological and Immunohistochemical Analysis of Pancreatic Tissue

Pancreata were excised from mice, and fixed overnight in neutral-buffered formalin, embedded in paraffin wax, and sectioned (5–10 µm). Frozen sections were prepared from tissue fixed in 4% paraformaldehyde for 2 hr, transferred to 30% sucrose in PBS overnight at 4°C, embedded in OCT, and frozen in foil on a bath of dry ice and ethanol. The pancreata sectioned (5–10 µm) across the whole block with 100 µm interval. All section were blocked by 10% BSA for 1 hour and incubated with following primary antibodies: Guinea pig anti-swine Insulin (1∶100 dilutions, Dako), Rabbit anti-human Glucagon (1∶100 dilutions Dako) and stained with following fluorescence probes: Alexa 633 goat anti-Guinea pig (1∶200 dilutions, Invitrogen), FITC goat anti-rabbit (1∶200 dilutions, Vector) and DAPI (Vector H-1200) in 5 levels each.

### Quantification of Beta Cells

Five levels through the whole pancreas were studied for each individual animal under confocal microscopy and images were captured by scanning each whole section blindly (whole pancreas under magnification of 50× and islets captured under 400×). Software Image J (National Institutes of Health, Bethesda, MD; http://rsb.info.nih.gov/ij/) was used for calculating each channel: insulin, or nuclear staining. Beta cell mass was calculated by dividing cross sectional area of insulin staining by whole section area and then multiplying by pancreas weight. Total beta cell number for the same 5 levels was calculated from captured images by using dedicated software developed for the purpose of segmentation and semi-automated cell classification and quantification [30].

### Statistics

Results between groups were compared by paired t test, with a p value<0.05 considered as achieving statistical significance and marked by *. All results were reported as the Means ± SEM.

## Supporting Information

Table S1 **Raw Data of Beta Cell Mass (mg) in MIG and MIGKO Mice.**
(DOC)

Table S2 **Raw Data of Beta Cell Number in MIG and MIGKO Mice (from 5 pancreas sections per mouse).**
(DOC)
