# Supplementary material for: Re-Expression of IGF-II Is Important for Beta Cell Regeneration in Adult Mice
Source: PLoS One. 2012 Sep 7;7(9):e43623. doi: 10.1371/journal.pone.0043623 (PMC3436856; doi:10.1371/journal.pone.0043623)
Supplement: Table S1 — Raw Data of Beta Cell Mass (mg) in MIG and MIGKO Mice. (DOC) [file pone.0043623.s001.doc]

**Table S1**

|  | **Control** | **Day 11 ablation** | **4 days recovery** | **3 months recovery** |
| --- | --- | --- | --- | --- |
| **MIG** | 2.390 | 0.473 | 0.814 | 2.873 |
|  | 10.039 | 0.373 | 0.912 | 3.470 |
|  | 7.283 | 0.373 | 0.744 | 1.321 |
| **MIGKO** | 1.297 | 0.568 | 0.283 | 2.852 |
|  | 1.200 | 0.114 | 0.202 | 0.901 |
|  | 3.407 | 0.217 | 0.332 | 1.498 |
