# Supplementary material for: Re-Expression of IGF-II Is Important for Beta Cell Regeneration in Adult Mice
Source: PLoS One. 2012 Sep 7;7(9):e43623. doi: 10.1371/journal.pone.0043623 (PMC3436856; doi:10.1371/journal.pone.0043623)
Supplement: Table S2 — Raw Data of Beta Cell Number in MIG and MIGKO Mice (from 5 pancreas sections per mouse). (DOC) [file pone.0043623.s002.doc]

**Table S2**

|  | **Control** | **Day 11 ablation** | **4 days recovery** | **3 months recovery** |
| --- | --- | --- | --- | --- |
| **MIG** | 14619 | 2392 | 3564 | 13006 |
|  | 20019 | 3301 | 5944 | 13396 |
|  | 12193 | 1459 | 1924 | 4139 |
| **MIGKO** | 9025 | 2832 | 2094 | 9993 |
|  | 11829 | 1548 | 1442 | 4487 |
|  | 23700 | 1420 | 1382 | 3770 |
